# Role of inflammation in túbulo-interstitial damage associated to obstructive nephropathy

**DOI:** 10.1186/1476-9255-7-19

**Published:** 2010-04-22

**Authors:** María T Grande, Fernando Pérez-Barriocanal, José M López-Novoa

**Affiliations:** 1 Instituto "Reina Sofía" de Investigación Nefrológica, Departamento de Fisiología y Farmacología, Universidad de Salamanca, Salamanca, Spain; 2 Red Cooperativa de Investigación Renal del Instituto Carlos III (RedinRen). Salamanca, Spain

## Abstract

Obstructive nephropathy is characterized by an inflammatory state in the kidney, that is promoted by cytokines and growth factors produced by damaged tubular cells, infiltrated macrophages and accumulated myofibroblasts. This inflammatory state contributes to tubular atrophy and interstitial fibrosis characteristic of obstructive nephropathy. Accumulation of leukocytes, especially macrophages and T lymphocytes, in the renal interstitium is strongly associated to the progression of renal injury. Proinflammatory cytokines, NF-κB activation, adhesion molecules, chemokines, growth factors, NO and oxidative stress contribute in different ways to progressive renal damage induced by obstructive nephropathy, as they induce leukocytes recruitment, tubular cell apoptosis and interstitial fibrosis. Increased angiotensin II production, increased oxidative stress and high levels of proinflammatory cytokines contribute to NF-κB activation which in turn induce the expression of adhesion molecules and chemokines responsible for leukocyte recruitment and iNOS and cytokines overexpression, which aggravates the inflammatory response in the damaged kidney. In this manuscript we revise the different events and regulatory mechanisms involved in inflammation associated to obstructive nephropathy.

## Introduction

Obstructive nephropathy due to congenital or acquired urinary tract obstruction is the first primary cause of chronic renal failure (CRF) in children, according to data of The North American Pediatric Renal Transplant Cooperative Study (NAPRTCS) [1]. Obstructive nephropathy is also a major cause of renal failure in adults [2,3].

The renal consequences of chronic urinary tract obstruction are very complex, and lead to renal injury and renal insufficiency. The experimental model of unilateral ureteral obstruction (UUO) in rat and mouse has become the standard model to understand the causes and mechanisms of nonimmunological tubulointerstitial fibrosis. This is because it is normotensive, nonproteinuric, nonhyperlipidemic, and without any apparent immune or toxic renal insult. The UUO consists of an acute obstruction of one of the ureter that mimics the different stages of obstructive nephropathy leading to tubulointerstitial fibrosis without compromising the life of the animal, because the contralateral kidney maintains or even increases its function due to compensatory functional and anatomic hypertrophy [2,3].

The evolution of renal structural and functional changes following urinary tract obstruction in these models has been well described. The first changes observed in the kidney are hemodynamic, beginning with renal vasoconstriction mediated by increased activity of the renin-angiotensin system and other vasoconstrictor systems [4]. Epithelial tubular cells are damaged by the stretch secondary to tubular distension and the increased hydrostatic pressure into the tubules due to accumulation of urine in the pelvis and the retrograde increase of interstitial pressure. This is followed by an interstitial inflammatory response initially characterized by macrophage infiltration. There is also a massive myofibroblasts accumulation in the interstitium. These myofibroblasts are formed by proliferation of resident fibroblasts, from bone marrow-derived cells, from pericyte infiltration, as well by epithelial-mesenchymal transformation (EMT), a complex process by which some tubular epithelial cells acquire mesenchymal phenotype and become activated myofibroblasts [5,6].

Damaged tubular cells, interstitial macrophages and myofibroblasts produce cytokines and growth factors that promote an inflammatory state in the kidney, induce tubular cell apoptosis and provoke the accumulation of extracellular matrix. The end-result of severe and chronic obstructive nephropathy is a progressive renal tubular atrophy with loss of nephrons accompanied by interstitial fibrosis. Thus, interstitial fibrosis is the result of these processes in a progressive and overlapping sequence. The evolution of renal injury in obstructive nephropathy shares many features with other forms of interstitial renal disease such as acute renal failure, polycystic kidney disease, aging kidney and renal transplant rejection [7-9]. The final fibrotic phase is very similar to virtually all progressive renal disorders, including glomerular disorders and systemic diseases such as diabetes or hypertension [4].

In this review we will analyze the role of inflammation on renal damage associated to obstructive nephropathy, and the cellular and molecular mechanisms involved in the genesis of these processes. As later described, the inflammatory process, through the release of cytokines and growth factors, results in the accumulation of interstitial macrophages which, in turn, release more cytokines and growth factors that contribute directly to tubular apoptosis and interstitial fibrosis [10,11].

### Urinary obstruction induces an inflammatory state in the kidney

In Sprague-Dawley rats subjected to chronic neonatal UUO (from 2 to 12 days), microarray analysis revealed that the mRNA expression of multiple immune modulators, including krox24, interferon-gamma regulating factor-1 (IRF-1), monocyte chemoattractant protein-1 (MCP-1), interleukin-1β (IL-1β), CCAAT/enhancer binding protein (C/EBP), p21, c-fos, c-jun, and pJunB, were significantly increased in obstructed compared to sham-operated kidneys, thus suggesting that UUO induces a pro-inflammatory environment [12]. This environment is characterized by up-regulation of inflammatory cytokines and factors that favors leukocyte infiltration. Other cytokines with different functions are also differentially regulated after UUO, and will contribute to the regulation of inflammation and interstitial infiltration. Thus, we will review the data available about the mechanisms involved in this inflammatory state, including nuclear factor κB (NF-κB) activation, increased oxidative stress, interstitial cell infiltration, and production of proinflammatory cytokines and other growth factors with inflammatory or anti-inflammatory properties, in the renal damage after UUO.

Thus, monocytes/macrophages, T cells, dendritic cells and neutrophils are involved in this inflammatory state of the kidney after UUO. Whereas interstitial macrophages increases 4 hours after UUO and constitute the predominant infiltrating cell population in acutely obstructed kidneys, T cells are also evident after 24 h of obstruction although neither B lymphocytes nor neutrophils are observed. Moreover, interstitial macrophages increases biphasically with an initial rapid increase during the first 24 h after UUO and the second phase following 72 h after UUO and all reports which observed an inverse correlation between interstitial macrophage number and the degree of fibrosis was noted at the later stage of UUO (day 14) and therefore it will be believed the possible renoprotective role for macrophages that infiltrate in the later phase after UUO [13].

### NF-κB activation

**Figure 1:**
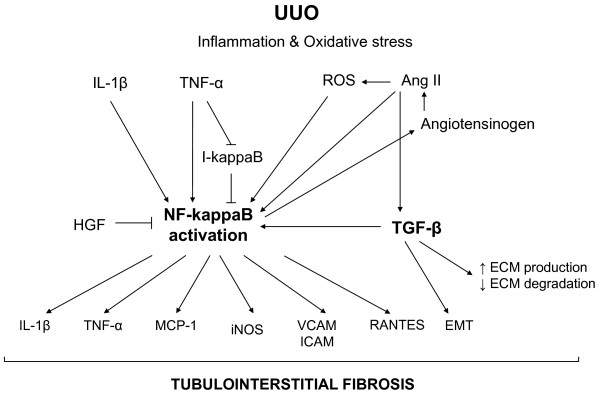
**Schematic representation of some of the signaling intermediates potentially involved in regulation of inflammatory response after UUO**. UUO induces IL-1β and TNF-α expression, leading to NF-κB activation. UUO also induces both oxidative stress and increased Angiotensin II (Ang II) levels. Ang II also activate the transcription factor NF-κB, both directly and indirectly, by promoting oxidative stress, which in turns activate Ang II by regulating angiotensinogen expression. TGF-β activates NF-κB through I-κB inhibition, a mechanism shared by TNF-α. NF-κB activation concludes in IL-1β and TNF-α expression enhancing NF-κB activation. Also NF-κB controls the expression of genes encoding pro-inflammatory cytokines, adhesion molecules and iNOS.

NF-κB is a ubiquitous and well-characterized transcription factor with a pivotal role in control of the inflammation, among other functions. Thus, NF-κB controls the expression of genes encoding pro-inflammatory cytokines (e. g., IL-1, IL-2, IL-6, TNF-α, etc.), chemokines (e. g., IL-8, MIP-1 α, MCP-1, RANTES, eotaxin, etc.), adhesion molecules (e. g., ICAM, VCAM, E-selectin), inducible enzymes (COX-2 and iNOS), growth factors, some of the acute phase proteins, and immune receptors, all of which play critical roles in controlling most inflammatory processes [14,15]. Also the PI3K/Akt pathway, which has been reported to be activated very early after UUO [16], results in activation of NF-κB [17]. NF-κB also controls the expression of EMT inducers (e.g., Snail1), and enhances EMT of mammary epithelial cells [18,19] (Figure 1).

NF-κB is activated by several cytokines such as IL-1β, TNF-α, by oxidative stress and by other molecules such as Angiotensin II (Ang II) [20]. Obstructed kidneys presented many cells that contained activated NF-κB complexes, in glomeruli, in tubulointerstitial cells and in infiltrating cells [21]. NF-κB is activated very early following UUO [22] and it is maintained activated during at least 7 days after UUO [21]. Furthermore, inhibition of NF-κB activation decreases apoptosis and interstitial fibrosis in rats with UUO [23]. NF-κB inhibition also diminishes monocyte infiltration and inflammation gene overexpression after UUO [21]. The administration of a proteasome inhibitor to maintain levels of I-κB, an endogenous inhibitor of NF-κB, reduces renal fibrosis and macrophage influx following UUO [24].

Renal cortical TNF-α levels increases early after UUO, whereas TNF-α neutralization with a pegylated form of soluble TNF receptor type 1 significantly reduced obstruction-induced TNF-α production, as well as NF-κB activation, IκB degradation, angiotensinogen expression, and renal tubular cell apoptosis, thus suggesting a major role for TNF-α in activating NF-κB via increased IκB-alpha phosphorylation [25].

In addition, curcumin, a phenolic compound with anti-inflammatory properties, has revealed protective action against interstitial inflammation in obstructive nephropathy by inhibition of the NF-κB-dependent pathway [26]. HGF has also been reported to inhibit renal inflammation, proinflammatory chemokine expression and renal fibrosis in an UUO model. The anti-inflammatory effect of HGF is mediated by disrupting nuclear factor NF-κB signaling, as later will be described [27].

NF-κB can be also activated by oxidative stress. The administration of antioxidant peptides to rats that suffered UUO was associated to a lower activation of NF-κB, and significantly attenuated the effects of ureteral obstruction on all aspects of renal damage associated to UUO [28]. Thus, oxidative stress seems to play also a major role in the UUO-associated inflammation.

### Oxidative stress

Oxidative stress has been implicated in the pathogenesis of various forms of renal injury [29]. Oxidative stress is also a major activator of the NF-κB and thus, an inductor of the inflammatory state [30] (Figure 1). There are several evidences showing that increased oxidative stress is involved in renal inflammatory damage after UUO. Reactive oxygen species are significantly increased in the chronically obstructed kidney [31] and a positive correlation was observed between the levels of free radical oxidation markers in the obstructed kidney tissue and in plasma [32]. Superoxide anion and hydrogen peroxide production increase significantly in the obstructed kidney [33]. After 5 days of obstruction, it has been reported a slight increase on renal cortex NADPH oxidase activity (a major source for superoxide production) whereas after 14 days of obstruction, a marked increase on NADPH oxidase activity was observed. In addition, decreased superoxide dismutase activity were demonstrated following 14 days of obstruction whereas no differences were noticed after 5 days of kidney obstruction [34].

Increased Ang II production, accumulation of activated phagocytes in the interstitial space and elevation of medium-weight molecules have been involved as responsible for the increased oxidative stress [35] after UUO. UUO also generate increased levels of carbonyl stress, and subsequently advanced glycation end-products (AGEs), and nitration adduct residues, both contributing to the progression of renal disease in the obstructed kidney [36,37]. The products of lipid peroxidation have been also found increased in both plasma and obstructed kidney after UUO [38]. Carboxymethyl-lysine, a marker for accumulated oxidative stress, was found to be increased in the interstitium of the obstructed kidneys [39]. Furthermore, heme oxygenase-1 (HO-1) expression, a sensitive indicator of cellular oxidative stress, was also found to be induced as early as 12 hours after ureteral obstruction [39]. All these results suggest that oxidative stress is involved in the pathogenesis of UUO. On the other hand, levels of the antioxidant enzyme catalase and copper-zinc superoxide dismutase, which prevent free radical damage, are lower in the obstructed kidney compared with the contralateral unobstructed kidney [33].

Antioxidant compounds, such as tocopherols reduce the level of oxidative stress observed after UUO [38]. Moreover, the administration of isotretinoin, a retinoid agonist, reduces renal macrophage infiltration in rats with UUO [39]. It should be noted that an increase in cellular reactive oxygen species (ROS) production stimulate the expression of the transcription factor Snail and favors EMT [40].

In short, oxidative stress markers levels increase in the kidney during UUO whereas levels of enzymes that prevent the oxidative damage are diminished in the obstructed kidney. All these data suggest that oxidative stress is increased in the obstructed kidney, and that increased oxidative stress plays a role in inducing an inflammatory state and in deteriorating the renal function of the obstructed kidney.

### Angiotensin II

Angiotensin II (Ang II) behaves in the kidney as a proinflammatory mediator, as it regulates a number of genes associated with progression of renal disease. The regulation of gene expression by Ang II occurs through changes in the activity of transcription factors within the nucleus of target cells. In particular, several members of the NF-κB family of transcription factors are activated by Ang II, which in turn fuels at least two autocrine reinforcing loops that amplify Ang II and TNF-α formation [41]. Thus, it is not surprisingly the interrelation between Ang II and proinflammatory cytokines effects in the interstitial cell infiltration after UUO. Many studies have demonstrated that obstructive nephropathy leads to activation of the intrarenal renin-angiotensin system [4,42,43]. This system is also activated in animal models of UUO. Ang II has a central role in the beginning and progression of obstructive nephropathy, both directly and indirectly, by stimulating production of molecules that contribute to renal injury. Following UUO, Ang II activates NF-κB, and the subsequent increased expression of proinflammatory genes [22]. In turn, the angiotensinogen gene is stimulated by activation of NF-κB [44] (Figure 1). In relation to the inflammatory process, Ang II type 1 receptor (AT1R) regulates several proinflammatory genes, including cytokines (interleukin-6 [IL-6]), chemokines (monocyte chemoattractant protein 1 [MCP-1]), and adhesion molecules (vascular cell adhesion molecule 1 [VCAM-1]) [45], but others, as the chemokine RANTES, are regulated by the Ang II type 2 receptor (AT2R) [46]. Some evidence suggests that AT2R participates in the inflammatory response in renal and vascular tissues [45-47]. *In vivo *and *in vitro *studies have shown that Ang II activates NF-κB in the kidney, *via *both AT1R and AT2R receptors [48,49].

Most studies have focused on the role of AT1R activation on kidney inflammation after UUO. For instance, inhibition or inactivation of AT1R also reduces NF-κB activation in the obstructed kidneys after UUO [50,51]. Also AT1R blockade, partially decreased macrophage infiltration in the obstructed kidney [21,50,52]. Thus AT1R activation seems to play a role in the UUO-associated inflammation. However, obstructed kidney in AT1R KO mice showed interstitial monocyte infiltration and NF-κB activation, and both processes were abolished by AT2R blockade, suggesting that AT2R activation plays also a major role in UUO-induced renal inflammation [21]. Simultaneous blockade of both AT1R and AT2R is able to completely prevent the inflammatory process after UUO [21], thus giving a further proof of the role of both receptors in the inflammatory state occurring after UUO. It should be noted that in wild-type mice reconstituted with bone marrow cells lacking the angiotensin AT1R gene, UUO results in more severe interstitial fibrosis despite fewer interstitial macrophages [53]. This effect seems to be due to impaired phagocytic function of AT1R-deficient macrophages [53]. This is a typical example of the fact that manipulation of a single molecule affecting more than one renal compartment could have opposite effects in different compartments.

Treatment with angiotensin converting enzyme (ACE) inhibitors greatly reduced the monocyte/macrophage infiltration in the obstructed kidney [54] but this reduction seems to be observed only in the short-term UUO, and 14 days after UUO ACE inhibitors did not decreased monocyte/macrophage infiltration, maybe because in late-stage UUO, infiltration is dependent on cytokines formation that is independent of Ang II [55].

Ang II also stimulates the activation of the small GTPase Rho, which in turn activates Rho-associated coiled-coil forming protein kinase (ROCK). Furthermore, inhibition of ROCK in mice with UUO significantly reduces macrophage infiltration and interstitial fibrosis [56].

### Proinflammatory cytokines in urinary obstruction

#### TNF-α and IL-1

The prototypical pro-inflammatory cytokines, TNF-α and interleukin-1 (IL-1), play a major role in the recruitment of inflammatory cells in the obstructed kidney [57-59]. Both TNF-α [60] and IL-1 [12,49] expression have been found augmented after renal obstruction. TNF-alpha production localized primarily to renal cortical tubular cells following obstruction [61] and dendritic cells [62]. The synthetic vitamin D analogue paricalcitol reduced infiltration of T cells and macrophages accompanied by a decreased expression of TNF-α in the obstructed kidney [63] and TNF-α neutralization reduced the degree of apoptotic renal tubular cell death although it did not prevent renal apoptosis completely, suggesting that other signaling pathways may contribute to obstruction-induced renal cell apoptosis [60]. The IL-1 receptor antagonist (IL-1ra) administration in mice with UUO inhibited IL-1 activity and subsequently decreased the infiltration of macrophages, the expression of ICAM-1 and the presence of alpha-smooth muscle actin (a marker of myofibroblasts) [59].

#### Other proinflammatory cytokines

Macrophage migratory inhibitory factor (MIF) is a proinflammatory cytokine which regulates leukocyte activation and fibroblast proliferation but although it is increased in the obstructed kidney after ureteral obstruction, MIF deficiency did not affect interstitial macrophage and T cell accumulation induced by UUO [64], thus suggesting that there are other factors that are also involved.

### Interstitial cell infiltration

It is now generally accepted that leukocyte infiltration and activation of interstitial macrophages play a central role in the renal inflammatory response to UUO [10]. The progression of renal injury in the obstructive nephropathy is closely associated with accumulation of leukocytes and fibroblasts in the damaged kidney. Leukocyte infiltration, especially macrophages and T lymphocytes, increases as early as 4 to 12 hours after ureteral obstruction and continues to increase over the course of days thereafter [65]. There are studies suggesting that lymphocyte infiltration does not seem to be required for progressive tubulointerstitial injury since immunocompromised mice with very low numbers of circulating lymphocytes showed the same degree of kidney damage after UUO [66]. However, macrophages are involved in the obstructed pathology [65,67] and macrophage secretion of galectin-3, a member of a large family of β-galactoside-binding lectins, is the major mechanism for macrophage to induce TGF-β-mediated myofibroblast activation and extracellular matrix production [68]. Macrophages can be functionally distinguished into two phenotypes based on cell surface markers and cytokine profile, M1 and M2 macrophages, suggesting different roles of macrophages in inflammation and tissue fibrosis [69]. Thus, whereas M1 macrophages produce MMPs and induce myofibroblasts to produce MMPs, M2 macrophages produce large amounts of TGF-β. It has been suggested that M1 macrophages may alter the equilibrium towards degradation during the later stages of fibrosis and play an important anti-fibrotic role [13].

Also, mast cells seem to protect the kidney against fibrosis by modulation of inflammatory cell infiltration as, after UUO, obstructed kidneys from mice deficient in mast cells showed increased fibrosis and infiltration of ERHR3-positive macrophages and CD3-positive T cells [70]. In a neonatal model of UUO in mice, blocking leukocyte recruitment by using the CCR-1 antagonist BX471 protected against tubular apoptosis and interstitial fibrosis, as evidenced by reduced monocyte influx, decreased EMT, and attenuated collagen deposition [71]. In this model, EMT was rapidly induced within 24 hours after UUO along with up-regulation of the transcription factors Snail1 and Snail2/Slug, preceding the induction of α-smooth muscle actin and vimentin. In the presence of BX471, the expression of chemokines, as well as of Snail1 and Snail2/Slug, in the obstructed kidney was completely attenuated. This was associated with reduced macrophage and T-cell infiltration, tubular apoptosis, and interstitial fibrosis in the developing kidney. These findings provide evidence that leukocytes induce EMT and renal fibrosis after UUO [71].

The recruitment of leukocytes from the circulation is mediated by several mechanisms including the activation of adhesion molecules, chemoattractant cytokines and proinflammatory and profibrotic mediators. Renal infiltrating cells have been characterized and quantitatively analyzed using specific blockers. For example, administration of liposome condronate deleted F4/80-possitive macrophages in mice and found that either F4/80+ monocytes/macrophages, F4/80+ dendritic cells, or both cell types contribute, at least in part, to the early development of renal fibrosis and tubular apoptosis [72]. These dendritic cells are considered an early source of proinflammatory mediators after acute UUO and play a specific role in recruitment and activation of effector-memory T-cells [62].

#### Adhesion molecules and leukocyte infiltration

Adhesion molecules are cell surface proteins involved in binding with other cells or with extracellular matrix. Adhesion molecules such as selectins, vascular cell adhesion molecule 1 (VCAM-1), intercellular adhesion molecule 1 (ICAM-1) and integrins plays a major role in leukocyte infiltration in several physiological and pathological conditions. We will next review their role in leukocyte recruitment after UUO.

##### Selectins

Selectins and their ligands mediate the initial contact between circulating leukocytes and the vascular endothelium resulting in capture and rolling of leukocytes along the vessel wall [73]. There are three different Selectins: E-selectin is expressed on endothelial cells, P-selectin on endothelial cells and platelets, and L-selectin on leukocytes. Whereas E-selectin expression is induced by inflammatory cytokines, P-selectin is rapidly mobilized to the surface of activated endothelium or platelets. L-selectin is constitutively expressed on most leukocytes. It has been reported that after ligation of the ureter, ligands for L-selectin rapidly disappeared from tubular epithelial cells and were relocated to the interstitium and peritubular capillary walls, where infiltration of monocytes and CD8(+) T cells subsequently occurred and mononuclear cell infiltration was significantly inhibited by neutralizing L-selectin, indicating the possible involvement of an L-selectin-mediated pathway [74]. In mice KO for P selectin, there is a marked decrease in macrophage infiltration in the obstructed kidney [75]. In other study using mice with a triple null mutation for E-, P-, and L-selectin (EPL^-/- ^mice), it has been reported that EPL^-/- ^mice compared with wild type mice, showed markedly lower interstitial macrophage infiltration, collagen deposition and tubular apoptosis after ureteral obstruction [76]. Furthermore, tubular apoptosis showed a significant correlation with macrophage infiltration [76]. Sulfatide, a sulphated glycolipid, is a L-selectin ligand in the rat kidney and contributes to the interstitial monocyte infiltration following UUO [77]. Sulfation of glycolipids is catalyzed by the enzime cerebroside sulfotransferase, and mice with a targeted deletion of this enzyme showed a considerable reduction in the number of monocytes/macrophages that infiltrated the interstitium after UUO. The number of monocytes/macrophages was also reduced to a similar extent in L-selectin KO mice, thus suggesting that sulfatide is a major L-selectin-binding molecule in the kidney and that the interaction between L-selectin and sulfatide plays a critical role in monocyte infiltration into the kidney interstitium alter UUO [77]

##### ICAM and VCAM

Vascular cell adhesion molecule 1 (VCAM-1) and intercellular adhesion molecule 1 (ICAM-1) plays a major role in firm leukocyte adherence to vessel wall, a prerequisite for leukocyte diapedesis. VCAM-1 and ICAM-1 involvement in obstructive nephropathy have been also studied. Both ICAM and VCAM expression was observed to be increased in the obstructed kidney, but with a different time course. ICAM expression increased as early as 3 hours [78] and continued high after 90 days of obstruction, while VCAM expression increased later, 2 or 3 days after obstruction [79,80]. Chronic UUO in weanling rats upregulated renal interstitial expression of ICAM-1 and macrophage-1 (Mac-1) antigen [81]. Both VCAM and ICAM immunostaining was higher in the expanding interstitium, but lower in glomeruli in obstructed kidney compared with contralateral kidneys, and only ICAM immunostaining within the apical tubular epithelium increase in both cortical and medullary cross-sections [78]. Inhibition of ICAM-1 by intravenous administration of antisense oligonucleotides against ICAM-1 markedly reduced interstitial inflammation and extracellular matrix following UUO in mice [82]. Inhibition of IL-1 by administration of genetically modified bone-marrow-derived vehicle cells containing an IL-1 receptor antagonist also reduced ICAM-1 expression and macrophage infiltration in mice with UUO [59], given a further support to the role of ICAM-1 expression as a key step in macrophage infiltration after UUO. No details of the role of PECAM in obstructive nephropathy have yet been reported to our knowledge.

##### Integrins and other molecules involved in leukocyte adhesion

Integrins are heterodimeric adhesion receptors consisting of noncovalently associated α and β subunits. β1-integrin interacts with LDL receptor-related protein 1 (LRP1) to mediate the activity of tPA as a fibrogenic cytokine in obstructed kidney [83]. Â2-integrins, mediate macrophage infiltration in obstructive nephropathy as targeted deletion of β2-integrins reduces early macrophage infiltration following UUO in the neonatal rat [84]. β2-integrins also mediate macrophage infiltration in obstructive nephropathy in weanling rats [81]. Also αvβ5 integrin interacts with the receptor for urokinase-type plasminogen activator (uPAR or CD87), which in response to ureteral obstruction was significantly upregulated [85], a finding consistent with the fact that obstructed kidneys from uPAR-/-mice showed lower leukocytes and macrophages recruitment in the interstitium than WT mice [85].

Other molecules that participate in leukocyte recruitment have been identified, including junctional adhesion molecules (JAMs) which engage interactions with leukocyte 1 and 2 integrins [86]. JAM-C recognizes macrophage-1 (Mac-1) antigen, a leukocyte integrin of particular interest because it has been reported to be the predominant leukocyte integrin involved in leukocyte recruitment after obstruction, and it is activated after UUO [81,84].

#### Chemokines involved in leukocyte infiltration

Infiltrating cells are attracted by chemokines following a concentration-dependent signal towards the source of chemokines. Chemokines are categorized into four groups depending on the spacing of their first two cysteine residues. Thus CC chemokines (or β-chemokines) have two adjacent cysteines near their amino terminal ends, whereas the two N-terminal cysteines of CXC chemokines (or α-chemokines) are separated by one amino acid, C chemokines (or γ chemokines) has only two cysteines; one N-terminal cysteine and one cysteine downstream. Finally CX3C chemokines (or δ-chemokines) have three amino acids between the two cysteines.

CC chemokines, MCP-1 (monocyte chemoattractant protein-1) and RANTES (Regulated on Activation Normal T cell Expressed and Secreted), have been reported to increase progressively from 2 to 10 days after UUO [67,87]. MCP-1 expression increases at 2 hours after obstruction, while RANTES and macrophage inflammatory protein 1 alpha (MIP-1α) expression are increased later, at day 5 after UUO [88]. Vielhauser et al. showed a prominent expression of MCP-1 mRNA in the interstitial mononuclear cell infiltrates and also cortical tubular epithelial cells of mouse obstructed kidney [89]. Intramuscular injection of a mutant MCP-1 gene can block macrophage recruitment and reduce renal fibrosis following UUO [90]. Upregulation of MCP-1, in turn, is suppressed by HO-1. Targeted deletion of HO-1 in other models of renal injury significantly increases MCP-1 expression [91].

CC chemokines receptors, CCR1, CCR2 and CCR5 have been reported to be overexpressed after UUO [87]. Moreover, studies in CCR1 KO mice revealed that deletion of the CCR1 receptor attenuates leukocyte recruitment following UUO [92]. Something similar occurred with the inhibition of the CCR1 receptor [93]. However, this did not occur with CCR5, suggesting that only CCR1 is required for leukocyte recruitment and fibrosis after UUO [92]. Targeted deletion of the CCR2 gene or administration of CCR2 inhibitors reduced macrophage infiltration and interstitial fibrosis following UUO [94].

The synthetic vitamin D analogue paricalcitol reduced infiltration of T cells and macrophages in the obstructed kidney accompanied by a decreased expression of RANTES [63].

CXC chemokines are also involved in leukocyte recruitment in UUO, as it has been reported that interferon-gamma-induced protein-10 (IP-10), a CXC chemokine that is a potent chemoattractant for activated T lymphocytes, natural killer cells, and monocytes is overexpressed in obstructed kidneys [95]. Its receptor, CXCR3 was also found to be upregulated after UUO [96]. Also, targeted deletion of its receptor, CXCR3, or administration of an anti-IP-10-neutralizing monoclonal antibody promoted renal fibrosis, without affecting macrophage or T cell infiltration in obstructed kidneys [96], thus suggesting that blockade of IP-10 via CXCR3 contributes to renal fibrosis, possibly by upregulation of transforming growth factor-beta1 (TGF-β1), concomitant with downregulation of hepatocyte growth factor (HGF). Thus, overexpression of IP-10 and CXCR3 after UUO seems to serve as a protective mechanism against renal fibrosis.

#### Growth factors involved in the regulation of leukocyte infiltration

Growth factors are proteins capable of regulating a variety of cellular processes and typically act as molecules carrying information between cells. In the setting of a pro-inflammatory situation, growth factors regulate several steps of the inflammatory process.

TGF-β1 is a pleiotropic cytokine involved in a wide range of pathophysiological processes. Many studies have reported an increase in TGF-β1 content after UUO [67]. There is no doubt that TGF-β1 plays a major role in stimulating ECM production after UUO. The profibrogenic effect of TGF-β1 is achieved by a combination of inhibition of the degradation of matrix proteins by increased generation of proteinase inhibitors and by decreased expression of degradative proteins such as collagenase. The net effect of TGF-β1 is extracellular matrix accumulation. Furthermore, TGF-β1 is a chemoattractant for fibroblasts, and also stimulates fibroblast proliferation. In addition, TGF-β1 is a major inducer of the transcription factor snail [97], and Snail overexpression in mice is sufficient to induce spontaneous renal fibrosis [98]. Experimental studies, in a variety of renal disorders, have shown that the sustained aberrant expression of renal TGF-β1 results in the pathological accumulation of extracellular matrix material in both the glomerulus and interstitial compartments. TGF-β expression has been found in macrophages [99] but its expression is stronger in renal tubular cells [100]

However this molecule has also several anti-inflammatory properties. First, TGF-β has opposing actions than those of the proinflammatory cytokines IL-1 and TNF-α in glomerular disease. Second, TGF-β is a prominent macrophage deactivator acting against macrophage-mediated kidney injury [101]. By the opposite, TGF-β is known to be a strong chemoattractant for monocytes [102]. In agreement with this property, a significant correlation between interstitial macrophage number and cortical TGF-β1 expression levels has been reported in the obstructed kidney [67]. The major origin of increase TGF-β1 levels after UUO seems to be the infiltrated macrophages [67]. Thus macrophage infiltration seems to play a major role in UUO-induced interstitial fibrosis. In a model of mice that overexpress latent TGF-β1 on skin, high levels of latent TGF-β1 shows renoprotective effects as mice are protected against renal inflammation after UUO. This protection seems to be mediated by upregulation of renal Smad7, an inhibitory Smad, which inhibits NF-κB activation by inducing IκB expression [103] (Figure 1). Leptin has been suggested as a cofactor of TGF-β activation in obstructed kidney after UUO and the blockade of leptin has been proposed as a therapeutic possibility to prevent or delay the fibrosis and inflammation observed in the obstructive nephropathy [104].

HGF is known to contribute to organogenesis and tissue repair through mitogenic, motogenic and morphogenic activities in the kidney [105]. Renal HGF levels increased rapidly after UUO, reaching a peak 3 days after obstruction. Seven days after UUO, HGF levels declined to half of those seen three days after UUO. Also the administration of exogenous HGF to mice with UUO produced a reduction in TGF-β levels that may be achieved, at least in part, by suppression of macrophage infiltration, as has been observed that HGF suppress infiltration of macrophages in the obstructive nephropathy [106,107]. HGF gene delivery inhibited interstitial infiltration of inflammatory T cells and macrophages, and suppressed expression of both RANTES and MCP-1 in a mouse model of obstructive nephropathy [27]. In contrast to several reports demonstrating that activation of PI3-kinase/Akt results in activation of NF-κB [17], this study indicates that PI3-kinase activation by HGF, through the phosphorylation and subsequent inactivation of GSK-3β, leads to the suppression of the NF-κB-mediated RANTES expression after UUO [27].

Paricalcitol, as noted above, reduced infiltration of T cells and macrophages in the obstructed kidney and the mechanism by which it works seems to be the inhibition of RANTES expression by promoting vitamin D receptor-mediated sequestration of NF-κB signaling [63].

The growth factor macrophage colony-stimulating factor-1 (M-CSF or CSF-1) is important in promoting monocyte survival and activation to macrophages and it is produced by tubular epithelial cells and fibroblasts, whereas macrophages generate inflammatory cytokines that are dependent on M-CSF. M-CSF expression is regulated by NF-κB activation [108] and it has been reported that M-CSF expression is increased in the obstructed kidneys after UUO and that this increase is correlated with the macrophage recruitment induced in the obstructed kidney [64,109]. Targeted deletion of M-CSF in mice with UUO reduced interstitial macrophage infiltration, proliferation and activation, and significantly diminished tubular apoptosis [110] thus suggesting the key role of M-CSF regulating damage induced by macrophages during UUO.

Agonists of the adenosine receptor transiently reduced renal macrophage infiltration and inflammation in ischemic renal injury [111] and its mechanism of action is probably related to the inhibition by adenosine of M-CSF, although this item is not yet completely proven [112]. However, adenosine receptor agonists do not reduce renal inflammation and injury after UUO [111].

#### Osteopontin and leukocyte infiltration in UUO

Osteopontin (OPN) is a tubular-derived glycoprotein with macrophage chemoattractant properties. Numerous studies have investigated the role of OPN in tubulointerstitial macrophage accumulation in the kidney [113,114]. Using OPN knockout mice, Persy et al. verified that OPN was a critical factor for interstitial macrophage accumulation after renal ischemia and reperfusion damage [115]. OPN is involved in the accumulation of macrophages within the renal cortex following UUO, as OPN expression increased 4-fold 1 day after UUO and persisted at this level for at least 5-days after UUO, and this increase was found to be correlated with interstitial macrophage infiltration [116,108]. Furthermore, targeted deletion of the OPN gene reduced macrophage infiltration and interstitial fibrosis in mice with UUO and enhanced tubular cells apoptosis. This suggests that OPN could play a different role in the tubular epithelial cells and the interstitium. Thus, OPN might contribute to renal interstitial injury and, at the same time, it might have a protective role on the tubular epithelial cells [117].

OPN is a major ligand of CD44 glycoproteins, and chronic UUO also increases tubular expression of the CD44 family of glycoproteins, which are generated by alternative splicing after transcription of a single gene. Targeted deletion of CD44 in mice with UUO reduces macrophage infiltration and interstitial fibrosis, but increases tubular apoptosis and tubular injury [118]. Thus, we can deduce that OPN has a dual role in obstructive nephropathy, with damaging effects on the renal interstitium and protective effects on the tubular epithelial cells.

**Figure 2:**
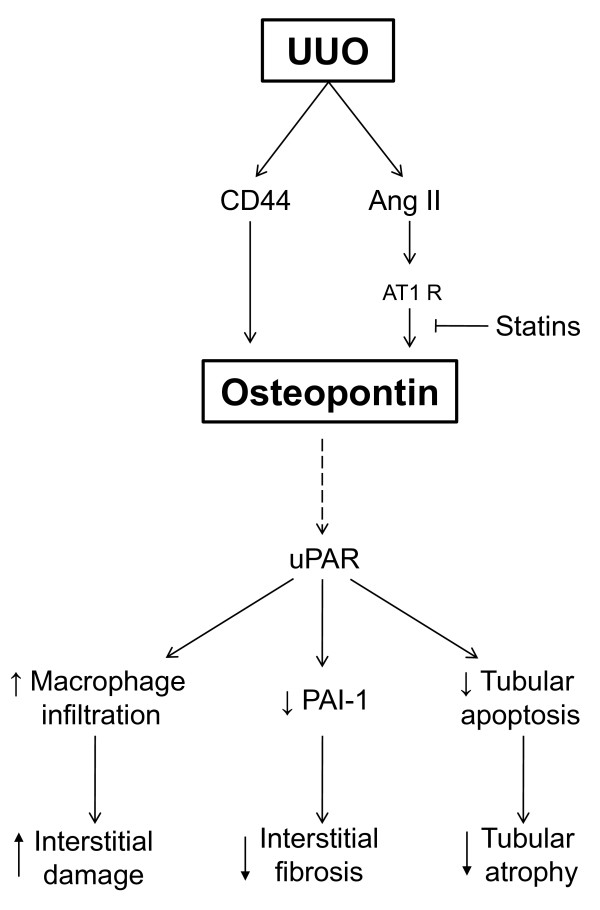
**Schematic illustration of the Osteopontin signaling pathway and effects during obstructive nephropathy**. UUO induces increased Angiotensin II (Ang II) levels which up-regulated Osteopontin (OPN) expression through AT1 receptor. This effect can be inhibited by statins. UUO also increases tubular expression of the CD44, a receptor of OPN. OPN actions may be mediated by uPAR, which reduces tubular apoptosis and interstitial fibrosis through reduced plasminogen activator inhibitor-1 (PAI-1) but promotes macrophage infiltration in the obstructive nephropathy. Discontinuous arrow connecting OPN and uPAR means that, although the relationship between them has been demonstrated "in vitro" (ref. 126 and 127), no direct relationship has been demonstrated in experimental or clinical models of obstructive nephropathy.

Ang II and losartan administration increased and decreased respectively OPN expression in the kidney, whereas angiotensinogen and AT1-receptor antisense inhibition inhibited OPN expression in tubular proximal cells [119,120]. This suggests that the increased levels of Ang II in the obstructed kidney, through AT1 receptor, up-regulated OPN expression and secretion by the proximal tubule, thus facilitating macrophage recruitment into the renal interstitium (Figure 2).

In UUO nephropathy, administration of simvastatin, a member of the HMG-CoA reductase inhibitors (statins) reduced renal inflammation, macrophage accumulation and fibrosis in tubulointerstitium, independent of their cholesterol-lowering effects [121]. Another statin, atorvastatin, reduced the number of macrophage on day 3 and on day 10 after UUO through downregulating the expression of OPN and M-CSF independent of cholesterol-lowering effects [108]. Statin-reduced OPN expression in UUO may also be related to its inhibiting effect on Ang II inflammatory effects on the kidney [122], as Ang II is a potent inducer of OPN [103]. On the other hand, statins also can inhibit NF-κB activation [123]. Furthermore, mizoribine, an immunosuppressive that inhibits selectively the proliferation of lymphocytes by interfering with inosine monophosphate dehydrogenase, inhibited the UUO-mediated OPN increment [124]. All these studies suggest a role of OPN in the leukocyte recruitment after ureteral obstruction. However Yoo et al. have found that the interstitial macrophage population did not differ in OPN null mutant (-/-) mice and WT mice after UUO [125] suggesting other roles for OPN during obstructive nephropathy. CD44 is one of the receptors of OPN and of hyaluronic acid and the CD44 expression is induced after UUO [118]. Moreover, obstructed kidneys from CD44^-/- ^mice subjected to UUO, showed lower macrophage infiltration than WT mice [118]. It has been also suggested that CD44 works as a facilitator of HGF signaling in vivo, as phosphorylation of c-Met, its high-affinity receptor, was attenuated in obstructed CD44^-/- ^kidneys, suggesting that CD44 is involved in the protective functions of HGF [118]. In addition, lower levels of OPN were observed in the obstructed kidney of urokinase receptor deficient mice (uPAR^-/-^) than in WT mice after UUO, thus suggesting that OPN-induced cell migration may be dependent on uPA-uPAR activity [85]. It should be noted that uPAR seems to play also a dual role on UUO-induced renal damage. Targeted deletion of uPAR in mice with UUO in one way reduces macrophage infiltration, but on the other hand increases accumulation of plasminogen activator inhibitor-1 (PAI-1) and interstitial fibrosis, as well as tubular apoptosis [85] (Figure 2). However it should be noted that although the connection between osteopontin and PAR has been reported in some "in vitro" studies [126,127], no reports on this connection has been published in experimental or clinical models or urinary obstruction.

### iNOS overexpression

Inducible nitric-oxide synthase (iNOS) overexpression is a characteristic hallmark of the inflammatory state and activation of the transcription factor NF-κB is thought to be essential for the induction of iNOS [128]. iNOS expression increases after UUO (Figure 1). Thus, 5 days after kidney obstruction there is an increased NO production and iNOS expression at transcriptional and post-transcriptional levels, whereas 14 days after obstruction, decreased endogenous NO production and lower iNOS expression at mRNA and protein levels were observed [34]. Tubular epithelial cells are most likely the major source of NO as these cells are subjected to a high pressure or mechanical stretch as a result of ureteral obstruction. When cultured tubular epithelial cells are subjected to high pressure (60 mmHg), there was an increase of iNOS expression, while endothelial NOS expression remained unchanged. Furthermore, the use of NF-κB inhibitors was shown to prevent the increase in iNOS expression, thus suggesting the role of this pro-inflammatory pathway in the iNOS overexpression [129]. In obstructed neonatal rats, *in vivo *administration of L-Arginine, which activates NO production by iNOS, prevented renal damage. Opposite effects were obtained after nitro L-Arginine methyl ester (L-NAME) treatment. These findings suggest that NO can produce resistance to obstruction-induced cell death in neonatal UUO [34]. Targeted deletion of inducible nitric oxide synthase (iNOS) in mice subjected to UUO increases renal macrophage infiltration and interstitial fibrosis, indicating that endogenous iNOS also serves to limit macrophage infiltration [130]. Administration of losartan to the UUO model in rats induced a down-regulation of iNOS, with persistent levels of eNOS in renal cortex of the obstructed kidney, thus suggesting that Ang II plays a major role in iNOS overexpression [131]. Liposome-mediated iNOS gene therapy improves renal function in rats with UUO [132] demonstrating that strategies to increase iNOS might be a powerful therapeutic approach in obstructive nephropathy [133].

## Conclusions and clinical perspectives

In this review we have summarized the most important factors that have been involved in the genesis and progression of the inflammatory damage induced by ureteral obstruction. These factors regulate cytokine and chemokines production, leukocyte/macrophage recruitment, interstitial inflammation, tubular cell apoptosis, and fibroblasts proliferation and activation (see table 1). NF-κB activation plays a central role in the inflammatory reaction after ureteral obstruction. Oxidative stress and renin-angiotensin II system seems to play a major role in activating NF-κB and they contribute also to the overexpression of pro-inflammatory cytokines in the obstructive nephropathy. As many therapeutic agents have been developed in the last years to control inflammation and NF-κB activation for the treatment of several diseases such as tumors [134], it can be postulated that this anti-inflammatory therapy could be useful to treat or prevent kidney damage during obstructive nephropathy [135]. There are many data in animal models, most of them reviewed in the present manuscript, demonstrating that anti-inflammatory treatment ameliorates renal damage in experimental models of obstructive nephropathy. Furthermore, attempts to avoid tubulointerstitial inflammation by immunosupression were successful to inhibit renal fibrosis. Rapamycin and mycophenolate mofetil (MMF), immunosuppressive agents, were described to improve the progression of injury elicited by UUO [136,137]. However, cost and adverse effects caused difficulty in the establishment of an efficient therapy based on that approach. It should be noted that clinical studies on these topics are almost absent in the literature. Thus, the anti-inflammatory therapy to treat obstructive nephropathy, although promising, needs many clinical studies that prove to be successful in the clinical setting.

**Table 1:** Summary effects of different molecules involved in inflammation in the obstructive nephropathy

Agent	Effect
**NF-κB**	Inflammatory gene expression Macrophage infiltration Renal tubular cell apoptosis

**Ang II**	NF-κB activation Oxidative stress TGF-β upregulation Macrophage infiltration

**TNF-α**	Macrophage infiltration Renal tubular cell death

**IL-1**	ICAM expression Macrophage infiltration Fibroblast activation

**MIF**	Leukocyte activation Fibroblast proliferation

**E,P,L Selectins**	Monocytes/macrophage and T cell infiltration Tubular apoptosis

**VCAM, ICAM**	Interstitial inflammation Leukocyte infiltration

**β-integrins**	Macrophage infiltration

**MCP-1, RANTES, MIP-1α**	Macrophage recruitment

**CCR1, CCR2**	Leukocyte recruitment Interstitial fibrosis

**JAMS**	Leukocyte recruitment

**M-CSF**	Macrophage infiltration, activation and proliferation Tubular apoptosis

**IP-10**	Leukocyte recruitment

**TGF-β**	Monocyte/macrophage infiltration Fibroblast proliferation Tubular apoptosis

**HGF**	Suppress macrophage infiltration Inhibit chemokine expression

**OPN**	Macrophage infiltration Interstitial fibrosis Repress tubular cell apoptosis

**iNOS**	Resistance to cell death Limit macrophage infiltration

## Competing interests

The authors declare that they have no competing interests.

## Authors' contributions

MTG and JML-N designed the review. MTG drafted the manuscript, FP-B and JML-N have rewritten the manuscript and MTG, FP-B and JML-N have completed the final version of the manuscript. All authors read and approved the final manuscript

## References

[B1] Smith JM Stablein DM Munoz R Hebert D McDonald RA Contributions of the Transplant Registry: The 2006 Annual Report of the North American Pediatric Renal Trials and Collaborative Studies (NAPRTCS) Pediatr Transplant 2007 11 366 373 10.1111/j.1399-3046.2007.00704.x 17493215

[B2] Klahr S Morrissey J Obstructive nephropathy and renal fibrosis Am J Physiol Renal Physiol 2002 283 F861 F875 12372761 10.1152/ajprenal.00362.2001

[B3] Klahr S Obstructive nephropathy Intern Med 2000 39 355 361 10.2169/internalmedicine.39.355 10830173

[B4] Chevalier RL Obstructive nephropathy: towards biomarker discovery and gene therapy Nat Clin Pract Nephrol 2006 2 157 168 10.1038/ncpneph0098 16932414

[B5] Iwano M Plieth D Danoff TM Xue C Okada H Neilson EG Evidence that fibroblasts derive from epithelium during tissue fibrosis J Clin Invest 2002 110 341 350 12163453 10.1172/JCI15518 PMC151091

[B6] Grande MT López-Novoa JM Fibroblast activation and myofibroblast generation in obstructive nephropathy Nat Rev Nephrol 2009 5 319 328 10.1038/nrneph.2009.74 19474827

[B7] Bohle A Muller GA Wehrmann M Mackensen-Haen S Xiao JC Pathogenesis of chronic renal failure in the primary glomerulopathies, renal vasculopathies, and chronic interstitial nephritides Kidney Int Suppl 1996 54 S2 S9 8731185

[B8] Ruiz-Torres MP Bosch RJ O'Valle F Del Moral RG Ramírez C Masseroli M Pérez-Caballero C Iglesias MC Rodríguez-Puyol M Rodríguez-Puyol D Age-related increase in expression of TGF-beta1 in the rat kidney: relationship to morphologic changes J Am Soc Nephrol 1998 9 782 791 9596075 10.1681/ASN.V95782

[B9] Paul LC Chronic allograft nephropathy: An update Kidney Int 1999 56 783 793 10.1046/j.1523-1755.1999.00611.x 10469349

[B10] Chevalier RL Pathogenesis of renal injury in obstructive uropathy Curr Opin Pediatr 2006 18 153 160 10.1097/01.mop.0000193287.56528.a4 16601495

[B11] Misseri R Meldrum KK Mediators of fibrosis and apoptosis in obstructive uropathies Curr Urol Rep 2005 6 140 145 10.1007/s11934-005-0083-5 15717973

[B12] Silverstein DM Travis BR Thornhill BA Schurr JS Kolls JK Leung JC Chevalier RL Altered expression of immune modulator and structural genes in neonatal unilateral ureteral obstruction Kidney Int 2003 64 25 35 10.1046/j.1523-1755.2003.00067.x 12787392

[B13] Nishida M Hamaoka K Macrophage phenotype and renal fibrosis in obstructive nephropathy Nephron Exp Nephrol 2008 110 e31 e36 10.1159/000151561 18724069

[B14] Nam NH Naturally occurring NF-kappaB inhibitors Mini Rev Med Chem 2006 6 945 951 10.2174/138955706777934937 16918500

[B15] Blackwell TS Christman JW The role of nuclear factor-kappa B in cytokine gene regulation Am J Respir Cell Mol Biol 1997 17 3 9 9224203 10.1165/ajrcmb.17.1.f132

[B16] Rodríguez-Peña AB Grande MT Eleno N Arévalo M Guerrero C Santos E López-Novoa JM Activation of Erk1/2 and Akt following unilateral ureteral obstruction Kidney Int 2008 74 196 209 10.1038/ki.2008.160 18449171

[B17] Ozes ON Mayo LD Gustin JA Pfeffer SR Pfeffer LM Donner DB NF-kappaB activation by tumour necrosis factor requires the Akt serine-threonine kinase Nature 1999 401 82 85 10.1038/43466 10485710

[B18] Julien S Puig I Caretti E Bonaventure J Nelles L van Roy F Dargemont C de Herreros AG Bellacosa A Larue L Activation of NF-kappaB by Akt upregulates Snail expression and induces epithelium mesenchyme transition Oncogene 2007 26 7445 7456 10.1038/sj.onc.1210546 17563753

[B19] Chua HL Bhat-Nakshatri P Clare SE Morimiya A Badve S Nakshatri H NF-kappaB represses E-cadherin expression and enhances epithelial to mesenchymal transition of mammary epithelial cells: potential involvement of ZEB-1 and ZEB-2 Oncogene 2007 26 711 724 10.1038/sj.onc.1209808 16862183

[B20] Bauge C Beauchef G Leclercq S Kim SJ Pujol JP Galéra P Boumédiene K NFkappaB mediates IL-1beta-induced down-regulation of TbetaRII through the modulation of Sp3 expression J Cell Mol Med 2008 12 1754 1766 10.1111/j.1582-4934.2007.00173.x 18053089 PMC3918091

[B21] Esteban V Lorenzo O Rupérez M Suzuki Y Mezzano S Blanco J Kretzler M Sugaya T Egido J Ruiz-Ortega M Angiotensin II, via AT1 and AT2 receptors and NF-kappaB pathway, regulates the inflammatory response in unilateral ureteral obstruction J Am Soc Nephrol 2004 15 1514 1529 10.1097/01.ASN.0000130564.75008.F5 15153562

[B22] Morrissey JJ Klahr S Enalapril decreases nuclear factor kappa B activation in the kidney with ureteral obstruction Kidney Int 1997 52 926 933 10.1038/ki.1997.414 9328931

[B23] Miyajima A Kosaka T Seta K Asano T Umezawa K Hayakawa M Novel nuclear factor kappa B activation inhibitor prevents inflammatory injury in unilateral ureteral obstruction J Urol 2003 169 1559 1563 10.1097/01.ju.0000045686.21766.c1 12629415

[B24] Tashiro K Tamada S Kuwabara N Komiya T Takekida K Asai T Iwao H Sugimura K Matsumura Y Takaoka M Nakatani T Miura K Attenuation of renal fibrosis by proteasome inhibition in rat obstructive nephropathy: possible role of nuclear factor kappaB Int J Mol Med 2003 12 587 592 12964039

[B25] Meldrum KK Metcalfe P Leslie JA Misseri R Hile KL Meldrum DR TNF-alpha neutralization decreases nuclear factor-kappaB activation and apoptosis during renal obstruction J Surg Res 2006 131 182 188 10.1016/j.jss.2005.11.581 16412467

[B26] Kuwabara N Tamada S Iwai T Teramoto K Kaneda N Yukimura T Nakatani T Miura K Attenuation of renal fibrosis by curcumin in rat obstructive nephropathy Urology 2006 67 440 446 10.1016/j.urology.2005.09.028 16461119

[B27] Giannopoulou M Dai C Tan X Wen X Michalopoulos GK Liu Y Hepatocyte growth factor exerts its anti-inflammatory action by disrupting nuclear factor-kappaB signaling Am J Pathol 2008 173 30 41 10.2353/ajpath.2008.070583 18502824 PMC2438283

[B28] Mizuguchi Y Chen J Seshan SV Poppas DP Szeto HH Felsen D A novel cell-permeable antioxidant peptide decreases renal tubular apoptosis and damage in unilateral ureteral obstruction Am J Physiol Renal Physiol 2008 295 F1545 F1553 10.1152/ajprenal.00395.2007 18784263 PMC2584902

[B29] Haugen E Nath KA The involvement of oxidative stress in the progression of renal injury Blood Purif 1999 17 58 65 10.1159/000014377 10449863

[B30] Heidland A Sebekova K Schinzel R Advanced glycation end products and the progressive course of renal disease Am J Kidney Dis 2001 38 S100 S106 10.1053/ajkd.2001.27414 11576932

[B31] Kawada N Moriyama T Ando A Fukunaga M Miyata T Kurokawa K Imai E Hori M Increased oxidative stress in mouse kidneys with unilateral ureteral obstruction Kidney Int 1999 56 1004 1013 10.1046/j.1523-1755.1999.00612.x 10469368

[B32] Klahr S Urinary tract obstruction Semin Nephrol 2001 21 133 145 10.1053/snep.2001.20942 11245776

[B33] Ricardo SD Ding G Eufemio M Diamond JR Antioxidant expression in experimental hydronephrosis: role of mechanical stretch and growth factors Am J Physiol 1997 272 F789 F798 9227641 10.1152/ajprenal.1997.272.6.F789

[B34] Manucha W Valles PG Cytoprotective role of nitric oxide associated with Hsp70 expression in neonatal obstructive nephropathy Nitric Oxide 2008 18 204 215 10.1016/j.niox.2008.01.005 18280260

[B35] Barinov EF Barabadze EV Zhdaniuk Iu I Dynamics and factors regulating the intensity of free radical processes during experimental supravesical block Vopr Med Khim 1992 38 5 7 1492399

[B36] Asami J Odani H Ishii A Oide K Sudo T Nakamura A Miyata N Otsuka N Maeda K Nakagawa J Suppression of AGE precursor formation following unilateral ureteral obstruction in mouse kidneys by transgenic expression of alpha-dicarbonyl/L-xylulose reductase Biosci Biotechnol Biochem 2006 70 2899 2905 10.1271/bbb.60311 17151462

[B37] Moriyama T Kawada N Nagatoya K Takeji M Horio M Ando A Imai E Hori M Fluvastatin suppresses oxidative stress and fibrosis in the interstitium of mouse kidneys with unilateral ureteral obstruction Kidney Int 2001 59 2095 2103 11380811 10.1046/j.1523-1755.2001.00724.x

[B38] Saborio P Krieg RJ Kuemmerle NB Norkus EP Schwartz CC Chan JC Alpha-tocopherol modulates lipoprotein cytotoxicity in obstructive nephropathy Pediatr Nephrol 2000 14 740 746 10.1007/PL00013428 10955918

[B39] Schaier M Jocks T Grone HJ Ritz E Wagner J Retinoid agonist isotretinoin ameliorates obstructive renal injury J Urol 2003 170 1398 1402 10.1097/01.ju.0000084620.64255.b3 14501777

[B40] Radisky DC Levy DD Littlepage LE Liu H Nelson CM Fata JE Leake D Godden EL Albertson DG Nieto MA Werb Z Bissell MJ Rac1b and reactive oxygen species mediate MMP-3-induced EMT and genomic instability Nature 2005 436 123 127 10.1038/nature03688 16001073 PMC2784913

[B41] Klahr S Morrissey J Comparative effects of ACE inhibition and angiotensin II receptor blockade in the prevention of renal damage Kidney Int Suppl 2002 S23 S26 12410850 10.1046/j.1523-1755.62.s82.5.x

[B42] Harris RC Martínez-Maldonado M Angiotensin II-mediated renal injury Miner Electrolyte Metab 1995 21 328 335 7565481

[B43] Chevalier RL Molecular and cellular pathophysiology of obstructive nephropathy Pediatr Nephrol 1999 13 612 619 10.1007/s004670050756 10460514

[B44] Klahr S Morrissey J Angiotensin II and gene expression in the kidney Am J Kidney Dis 1998 31 171 176 10.1053/ajkd.1998.v31.pm9428470 9428470

[B45] Ruiz-Ortega M Rupérez M Esteban V Rodríguez-Vita J Sánchez-López E Egido J Modulation of angiotensin II effects, a potential novel approach to inflammatory and immune diseases Curr Med Chem 2003 2 379 394

[B46] Wolf G Ziyadeh FN Thaiss F Tomaszewski J Caron RJ Wenzel U Zahner G Helmchen U Stahl RA Angiotensin II stimulates expression of the chemokine RANTES in rat glomerular endothelial cells. Role of the angiotensin type 2 receptor J Clin Invest 1997 100 1047 1058 10.1172/JCI119615 9276721 PMC508279

[B47] Akishita M Horiuchi M Yamada H Zhang L Shirakami G Tamura K Ouchi Y Dzau VJ Inflammation influences vascular remodeling through AT2 receptor expression and signaling Physiol Genomics 2000 2 13 20 11015577 10.1152/physiolgenomics.2000.2.1.13

[B48] Ruiz-Ortega M Lorenzo O Rupérez M Blanco J Egido J Systemic infusion of angiotensin II into normal rats activates nuclear factor-kappaB and AP-1 in the kidney: role of AT(1) and AT(2) receptors Am J Pathol 2001 158 1743 1756 11337372 10.1016/s0002-9440(10)64130-2 PMC1891960

[B49] Esteban V Rupérez M Vita JR López ES Mezzano S Plaza JJ Egido J Ruiz-Ortega M Effect of simultaneous blockade of AT1 and AT2 receptors on the NFkappaB pathway and renal inflammatory response Kidney Int Suppl 2003 S33 S38 10.1046/j.1523-1755.64.s86.7.x 12969125

[B50] Nakatani T Tamada S Asai T Iwai Y Kim T Tsujino T Kumata N Uchida J Tashiro K Kuwabara N Komiya T Sumi T Okamura M Miura K Role of renin-angiotensin system and nuclear factor-kappaB in the obstructed kidney of rats with unilateral ureteral obstruction Jpn J Pharmacol 2002 90 361 364 10.1254/jjp.90.361 12501014

[B51] Satoh M Kashihara N Yamasaki Y Maruyama K Okamoto K Maeshima Y Sugiyama H Sugaya T Murakami K Makino H Renal interstitial fibrosis is reduced in angiotensin II type 1a receptor-deficient mice J Am Soc Nephrol 2001 12 317 325 11158221 10.1681/ASN.V122317

[B52] Kellner D Chen J Richardson I Seshan SV El Chaar M Vaughan ED Jr Poppas D Felsen D Angiotensin receptor blockade decreases fibrosis and fibroblast expression in a rat model of unilateral ureteral obstruction J Urol 2006 176 806 812 10.1016/j.juro.2006.03.076 16813952

[B53] Nishida M Fujinaka H Matsusaka T Price J Kon V Fogo AB Davidson JM Linton MF Fazio S Homma T Yoshida H Ichikawa I Absence of angiotensin II type 1 receptor in bone marrow-derived cells is detrimental in the evolution of renal fibrosis J Clin Invest 2002 110 1859 1868 12488436 10.1172/JCI200215045 PMC151648

[B54] Ishidoya S Morrissey J McCracken R Reyes A Klahr S Angiotensin II receptor antagonist ameliorates renal tubulointerstitial fibrosis caused by unilateral ureteral obstruction Kidney Int 1995 47 1285 1294 10.1038/ki.1995.183 7637258

[B55] Turan T van Harten JG de Water R Tuncay OL Kok DJ Is enalapril adequate for the prevention of renal tissue damage caused by unilateral ureteral obstruction and/or hyperoxaluria? Urol Res 2003 31 212 217 10.1007/s00240-003-0320-7 12719949

[B56] Nagatoya K Moriyama T Kawada N Takeji M Oseto S Murozono T Ando A Imai E Hori M Y-27632 prevents tubulointerstitial fibrosis in mouse kidneys with unilateral ureteral obstruction Kidney Int 2002 61 1684 1695 10.1046/j.1523-1755.2002.00328.x 11967018

[B57] Hashem RM Soliman HM Shaapan SF Turmeric-based diet can delay apoptosis without modulating NF-kappaB in unilateral ureteral obstruction in rats J Pharm Pharmacol 2008 60 83 89 10.1211/jpp.60.1.0011 18088509

[B58] Metcalfe PD Leslie JA Campbell MT Meldrum DR Hile KL Meldrum KK Testosterone exacerbates obstructive renal injury by stimulating TNF-alpha production and increasing proapoptotic and profibrotic signaling Am J Physiol Endocrinol Metab 2008 294 E435 E443 10.1152/ajpendo.00704.2006 18073317

[B59] Yamagishi H Yokoo T Imasawa T Shen JS Hisada Y Eto Y Kawamura T Hosoya T Genetically modified bone marrow-derived vehicle cells site specifically deliver an anti-inflammatory cytokine to inflamed interstitium of obstructive nephropathy J Immunol 2001 166 609 616 11123344 10.4049/jimmunol.166.1.609

[B60] Misseri R Meldrum DR Dinarello CA Dagher P Hile KL Rink RC Meldrum KK TNF-alpha mediates obstruction-induced renal tubular cell apoptosis and proapoptotic signaling Am J Physiol Renal Physiol 2005 288 F406 F411 10.1152/ajprenal.00099.2004 15507546

[B61] Misseri R Meldrum DR Dagher P Hile K Rink RC Meldrum KK Unilateral ureteral obstruction induces renal tubular cell production of tumor necrosis factor-alpha independent of inflammatory cell infiltration J Urol 2004 172 1595 1599 10.1097/01.ju.0000138902.57626.70 15371768

[B62] Dong X Bachman LA Miller MN Nath KA Griffin MD Dendritic cells facilitate accumulation of IL-17 T cells in the kidney following acute renal obstruction Kidney Int 2008 74 1294 1309 10.1038/ki.2008.394 18974760 PMC2948974

[B63] Tan X Wen X Liu Y Paricalcitol inhibits renal inflammation by promoting vitamin D receptor-mediated sequestration of NF-kappaB signaling J Am Soc Nephrol 2008 19 1741 1752 10.1681/ASN.2007060666 18525004 PMC2518439

[B64] Rice EK Nikolic-Paterson DJ David JR Bucala R Metz CN Atkins RC Tesch GH Macrophage accumulation and renal fibrosis are independent of macrophage migration inhibitory factor in mouse obstructive nephropathy Nephrology (Carlton) 2004 9 278 287 10.1111/j.1440-1797.2004.00319.x 15504140

[B65] Diamond JR Macrophages and progressive renal disease in experimental hydronephrosis Am J Kidney Dis 1995 26 133 140 10.1016/0272-6386(95)90166-3 7611244

[B66] Shappell SB Gurpinar T Lechago J Suki WN Truong LD Chronic obstructive uropathy in severe combined immunodeficient (SCID) mice: lymphocyte infiltration is not required for progressive tubulointerstitial injury J Am Soc Nephrol 1998 9 1008 1017 9621283 10.1681/ASN.V961008

[B67] Diamond JR Kees-Folts D Ding G Frye JE Restrepo NC Macrophages, monocyte chemoattractant peptide-1, and TGF-beta 1 in experimental hydronephrosis Am J Physiol 1994 266 F926 F933 7517644 10.1152/ajprenal.1994.266.6.F926

[B68] Henderson NC Mackinnon AC Farnworth SL Kipari T Haslett C Iredale JP Liu FT Hughes J Sethi T Galectin-3 expression and secretion links macrophages to the promotion of renal fibrosis Am J Pathol 2008 172 288 298 10.2353/ajpath.2008.070726 18202187 PMC2312353

[B69] Lin SL Castaño AP Nowlin BT Lupher ML Jr Duffield JS Bone marrow Ly6Chigh monocytes are selectively recruited to injured kidney and differentiate into functionally distinct populations J Immunol 2009 183 6733 6743 10.4049/jimmunol.0901473 19864592

[B70] Kim DH Moon SO Jung YJ Lee AS Kang KP Lee TH Lee S Chai OH Song CH Jang KY Sung MJ Zhang X Park SK Kim W Mast cells decrease renal fibrosis in unilateral ureteral obstruction Kidney Int 2009 75 1031 1038 10.1038/ki.2009.1 19242503

[B71] Lange-Sperandio B Trautmann A Eickelberg O Jayachandran A Oberle S Schmidutz F Rodenbeck B Hömme M Horuk R Schaefer F Leukocytes induce epithelial to mesenchymal transition after unilateral ureteral obstruction in neonatal mice Am J Pathol 2007 171 861 871 10.2353/ajpath.2007.061199 17675578 PMC1959504

[B72] Kitamoto K Machida Y Uchida J Izumi Y Shiota M Nakao T Iwao H Yukimura T Nakatani T Miura K Effects of liposome clodronate on renal leukocyte populations and renal fibrosis in murine obstructive nephropathy J Pharmacol Sci 2009 111 285 292 10.1254/jphs.09227FP 19893275

[B73] Springer TA Traffic signals for lymphocyte recirculation and leukocyte emigration: the multistep paradigm Cell 1994 76 301 314 10.1016/0092-8674(94)90337-9 7507411

[B74] Shikata K Suzuki Y Wada J Hirata K Matsuda M Kawashima H Suzuki T Iizuka M Makino H Miyasaka M L-selectin and its ligands mediate infiltration of mononuclear cells into kidney interstitium after ureteric obstruction J Pathol 1999 188 93 99 10.1002/(SICI)1096-9896(199905)188:1<93::AID-PATH305>3.0.CO;2-# 10398147

[B75] Naruse T Yuzawa Y Akahori T Mizuno M Maruyama S Kannagi R Hotta N Matsuo S P-selectin-dependent macrophage migration into the tubulointerstitium in unilateral ureteral obstruction Kidney Int 2002 62 94 105 10.1046/j.1523-1755.2002.00419.x 12081568

[B76] Lange-Sperandio B Cachat F Thornhill BA Chevalier RL Selectins mediate macrophage infiltration in obstructive nephropathy in newborn mice Kidney Int 2002 61 516 524 10.1046/j.1523-1755.2002.00162.x 11849391

[B77] Ogawa D Shikata K Honke K Sato S Matsuda M Nagase R Tone A Okada S Usui H Wada J Miyasaka M Kawashima H Suzuki Y Suzuki T Taniguchi N Hirahara Y Tadano-Aritomi K Ishizuka I Tedder TF Makino H Cerebroside sulfotransferase deficiency ameliorates L-selectin-dependent monocyte infiltration in the kidney after ureteral obstruction J Biol Chem 2004 279 2085 2090 10.1074/jbc.M305809200 14583626

[B78] Shappell SB Mendoza LH Gurpinar T Smith CW Suki WN Truong LD Expression of adhesion molecules in kidney with experimental chronic obstructive uropathy: the pathogenic role of ICAM-1 and VCAM-1 Nephron 2000 85 156 166 10.1159/000045649 10867522

[B79] Morrissey JJ Klahr S Differential effects of ACE and AT1 receptor inhibition on chemoattractant and adhesion molecule synthesis Am J Physiol 1998 274 F580 F586 9530275 10.1152/ajprenal.1998.274.3.F580

[B80] Ricardo SD Levinson ME DeJoseph MR Diamond JR Expression of adhesion molecules in rat renal cortex during experimental hydronephrosis Kidney Int 1996 50 2002 2010 10.1038/ki.1996.522 8943483

[B81] Takeda A Fukuzaki A Kaneto H Ishidoya S Ogata Y Sasaki T Konda R Sakai K Orikasa S Role of leukocyte adhesion molecules in monocyte/macrophage infiltration in weanling rats with unilateral ureteral obstruction Int J Urol 2000 7 415 420 10.1046/j.1442-2042.2000.00222.x 11144652

[B82] Cheng QL Chen XM Li F Lin HL Ye YZ Fu B Effects of ICAM-1 antisense oligonucleotide on the tubulointerstitium in mice with unilateral ureteral obstruction Kidney Int 2000 57 183 190 10.1046/j.1523-1755.2000.00825.x 10620199

[B83] Hu K Wu C Mars WM Liu Y Tissue-type plasminogen activator promotes murine myofibroblast activation through LDL receptor-related protein 1-mediated integrin signaling J Clin Invest 2007 117 3821 3832 10.1172/JCI32405 18037995 PMC2082143

[B84] Lange-Sperandio B Schimpgen K Rodenbeck B Chavakis T Bierhaus A Nawroth P Thornhill B Schaefer F Chevalier RL Distinct roles of Mac-1 and its counter-receptors in neonatal obstructive nephropathy Kidney Int 2006 69 81 88 10.1038/sj.ki.5000017 16374427

[B85] Zhang G Kim H Cai X Lopez-Guisa JM Carmeliet P Eddy AA Urokinase receptor modulates cellular and angiogenic responses in obstructive nephropathy J Am Soc Nephrol 2003 14 1234 1253 10.1097/01.ASN.0000064701.70231.3F 12707393

[B86] Sircar M Bradfield PF Aurrand-Lions M Fish RJ Alcaide P Yang L Newton G Lamont D Sehrawat S Mayadas T Liang TW Parkos CA Imhof BA Luscinskas FW Neutrophil transmigration under shear flow conditions in vitro is junctional adhesion molecule-C independent J Immunol 2007 178 5879 5887 17442972 10.4049/jimmunol.178.9.5879

[B87] Vielhauer V Anders HJ Mack M Cihak J Strutz F Stangassinger M Luckow B Gröne HJ Schlöndorff D Obstructive nephropathy in the mouse: progressive fibrosis correlates with tubulointerstitial chemokine expression and accumulation of CC chemokine receptor 2- and 5-positive leukocytes J Am Soc Nephrol 2001 12 1173 1187 11373340 10.1681/ASN.V1261173

[B88] Kaneto H Fukuzaki A Ishidoya S Takeda A Ogata Y Sasaki T Yamada S Orikasa S mRNA expression of chemokines in rat kidneys with ureteral obstruction Nippon Hinyokika Gakkai Zasshi 2000 91 69 74 10723179 10.5980/jpnjurol1989.91.69

[B89] Vielhauer V Anders HJ Mack M Cihak J Strutz F Stangassinger M Luckow B Gröne HJ Schlöndorff D Obstructive nephropathy in the mouse: progressive fibrosis correlates with tubulointerstitial chemokine expression and accumulation of CC chemokine receptor 2- and 5-positive leukocytes J Am Soc Nephrol 2001 12 1173 1187 11373340 10.1681/ASN.V1261173

[B90] Wada T Furuichi K Sakai N Iwata Y Kitagawa K Ishida Y Kondo T Hashimoto H Ishiwata Y Mukaida N Tomosugi N Matsushima K Egashira K Yokoyama H Gene therapy via blockade of monocyte chemoattractant protein-1 for renal fibrosis J Am Soc Nephrol 2004 15 940 948 10.1097/01.ASN.0000120371.09769.80 15034096

[B91] Pittock ST Norby SM Grande JP Croatt AJ Bren GD Badley AD Caplice NM Griffin MD Nath KA MCP-1 is up-regulated in unstressed and stressed HO-1 knockout mice: Pathophysiologic correlates Kidney Int 2005 68 611 622 10.1111/j.1523-1755.2005.00439.x 16014038

[B92] Eis V Luckow B Vielhauer V Siveke JT Linde Y Segerer S Pérez De Lema G Cohen CD Kretzler M Mack M Horuk R Murphy PM Gao JL Hudkins KL Alpers CE Gröne HJ Schlöndorff D Anders HJ Chemokine receptor CCR1 but not CCR5 mediates leukocyte recruitment and subsequent renal fibrosis after unilateral ureteral obstruction J Am Soc Nephrol 2004 15 337 347 10.1097/01.ASN.0000111246.87175.32 14747380

[B93] Anders HJ Vielhauer V Frink M Linde Y Cohen CD Blattner SM Kretzler M Strutz F Mack M Gröne HJ Onuffer J Horuk R Nelson PJ Schlöndorff D A chemokine receptor CCR-1 antagonist reduces renal fibrosis after unilateral ureter ligation J Clin Invest 2002 109 251 259 11805137 10.1172/JCI14040 PMC150841

[B94] Kitagawa K Wada T Furuichi K Hashimoto H Ishiwata Y Asano M Takeya M Kuziel WA Matsushima K Mukaida N Yokoyama H Blockade of CCR2 ameliorates progressive fibrosis in kidney Am J Pathol 2004 165 237 246 15215179 10.1016/S0002-9440(10)63292-0 PMC1618531

[B95] Crisman JM Richards LL Valach DP Franzoni DF Diamond JR Chemokine expression in the obstructed kidney Exp Nephrol 2001 9 241 248 10.1159/000052618 11423723

[B96] Nakaya I Wada T Furuichi K Sakai N Kitagawa K Yokoyama H Ishida Y Kondo T Sugaya T Kawachi H Shimizu F Narumi S Haino M Gerard C Matsushima K Kaneko S Blockade of IP-10/CXCR3 promotes progressive renal fibrosis Nephron Exp Nephrol 2007 107 12 21 10.1159/000106505 17671396

[B97] Zavadil J Böttinger EP TGF-beta and epithelial-to-mesenchymal transitions Oncogene 2005 24 5764 5774 10.1038/sj.onc.1208927 16123809

[B98] Boutet A De Frutos CA Maxwell PH Mayol MJ Romero J Nieto MA Snail activation disrupts tissue homeostasis and induces fibrosis in the adult kidney EMBO J 2006 25 5603 5613 10.1038/sj.emboj.7601421 17093497 PMC1679761

[B99] Ma LJ Yang H Gaspert A Carlesso G Barty MM Davidson JM Sheppard D Fogo AB Transforming growth factor-beta-dependent and -independent pathways of induction of tubulointerstitial fibrosis in beta6(-/-) mice Am J Pathol 2003 163 1261 1273 14507636 10.1016/s0002-9440(10)63486-4 PMC1868298

[B100] Fukuda K Yoshitomi K Yanagida T Tokumoto M Hirakata H Quantification of TGF-beta1 mRNA along rat nephron in obstructive nephropathy Am J Physiol Renal Physiol 2001 281 F513 F521 11502600 10.1152/ajprenal.2001.281.3.F513

[B101] Kitamura M Suto TS TGF-beta and glomerulonephritis: anti-inflammatory versus prosclerotic actions Nephrol Dial Transplant 1997 12 669 679 10.1093/ndt/12.4.669 9140992

[B102] Wahl SM Hunt DA Wakefield LM McCartney-Francis N Wahl LM Roberts AB Sporn MB Transforming growth factor type β induces monocyte chemotaxis and growth factor production Proc Natl Acad Sci USA 1987 84 5788 5792 10.1073/pnas.84.16.5788 2886992 PMC298948

[B103] Wang W Huang XR Li AG Liu F Li JH Truong LD Wang XJ Lan HY Signaling mechanism of TGF-beta1 in prevention of renal inflammation: role of Smad7 J Am Soc Nephrol 2005 16 1371 1383 10.1681/ASN.2004121070 15788474

[B104] Kumpers P Gueler F Rong S Mengel M Tossidou I Peters I Haller H Schiffer M Leptin is a coactivator of TGF-beta in unilateral ureteral obstructive kidney disease Am J Physiol Renal Physiol 2007 293 F1355 F1362 10.1152/ajprenal.00003.2007 17686962

[B105] Balkovetz DF Lipschutz JH Hepatocyte growth factor and the kidney: it is not just for the liver Int Rev Cytol 1999 186 225 260 full_text 9770301 10.1016/s0074-7696(08)61055-4

[B106] Mizuno S Matsumoto K Nakamura T Hepatocyte growth factor suppresses interstitial fibrosis in a mouse model of obstructive nephropathy Kidney Int 2001 59 1304 1314 10.1046/j.1523-1755.2001.0590041304.x 11260391

[B107] Gao X Mae H Ayabe N Takai T Oshima K Hattori M Ueki T Fujimoto J Tanizawa T Hepatocyte growth factor gene therapy retards the progression of chronic obstructive nephropathy Kidney Int 2002 62 1238 1248 10.1111/j.1523-1755.2002.kid579.x 12234294

[B108] Wardle EN Nuclear factor kappaB for the nephrologist Nephrol Dial Transplant 2001 16 1764 1768 10.1093/ndt/16.9.1764 11522856

[B109] Tian S Ding G Jia R Chu G Tubulointerstitial macrophage accumulation is regulated by sequentially expressed osteopontin and macrophage colony-stimulating factor: implication for the role of atorvastatin Mediators Inflamm 2006 **2006: **Article ID 12919, 9 pages. 16883060 10.1155/MI/2006/12919 PMC1592581

[B110] Lenda DM Kikawada E Stanley ER Kelley VR Reduced macrophage recruitment, proliferation, and activation in colony-stimulating factor-1-deficient mice results in decreased tubular apoptosis during renal inflammation J Immunol 2003 170 3254 3262 12626584 10.4049/jimmunol.170.6.3254

[B111] Lange-Sperandio B Forbes MS Thornhill B Okusa MD Linden J Chevalier RL A2A adenosine receptor agonist and PDE4 inhibition delays inflammation but fails to reduce injury in experimental obstructive nephropathy Nephron Exp Nephrol 2005 100 e113 e123 10.1159/000085057 15824514

[B112] Xaus J Valledor AF Cardo M Marquès L Beleta J Palacios JM Celada A Adenosine inhibits macrophage colony-stimulating factor-dependent proliferation of macrophages through the induction of p27kip-1 expression J Immunol 1999 163 4140 4149 10510349

[B113] Panzer U Thaiss F Zahner G Barth P Reszka M Reinking RR Wolf G Helmchen U Stahl RA Monocyte chemoattractant protein-1 and osteopontin differentially regulate monocytes recruitment in experimental glomerulonephritis Kidney Int 2001 59 1762 1769 10.1046/j.1523-1755.2001.0590051762.x 11318946

[B114] Xie Y Sakatsume M Nishi S Narita I Arakawa M Gejyo F Expression, roles, receptors, and regulation of osteopontin in the kidney Kidney Int 2001 60 1645 1657 10.1046/j.1523-1755.2001.00032.x 11703581

[B115] Persy VP Verhulst A Ysebaert DK De Greef KE De Broe ME Reduced postischemic macrophage infiltration and interstitial fibrosis in osteopontin knockout mice Kidney Int 2003 63 543 553 10.1046/j.1523-1755.2003.00767.x 12631119

[B116] Kaneto H Morrissey J McCracken R Reyes A Klahr S Osteopontin expression in the kidney during unilateral ureteral obstruction Miner Electrolyte Metab 1998 24 227 237 10.1159/000057375 9554561

[B117] Ophascharoensuk V Giachelli CM Gordon K Hughes J Pichler R Brown P Liaw L Schmidt R Shankland SJ Alpers CE Couser WG Johnson RJ Obstructive uropathy in the mouse: role of osteopontin in interstitial fibrosis and apoptosis Kidney Int 1999 56 571 580 10.1046/j.1523-1755.1999.00580.x 10432396

[B118] Rouschop KM Sewnath ME Claessen N Roelofs JJ Hoedemaeker I Neut R van der Aten J Pals ST Weening JJ Florquin S CD44 deficiency increases tubular damage but reduces renal fibrosis in obstructive nephropathy J Am Soc Nephrol 2004 15 674 686 10.1097/01.ASN.0000115703.30835.96 14978169

[B119] Diamond JR Kreisberg R Evans R Nguyen TA Ricardo SD Regulation of proximal tubular osteopontin in experimental hydronephrosis in the rat Kidney Int 1998 54 1501 1509 10.1046/j.1523-1755.1998.00137.x 9844126

[B120] Ricardo SD Franzoni DF Roesener CD Crisman JM Diamond JR Angiotensinogen and AT(1) antisense inhibition of osteopontin translation in rat proximal tubular cells Am J Physiol Renal Physiol 2000 278 F708 F716 10807582 10.1152/ajprenal.2000.278.5.F708

[B121] Vieira JM Jr Mantovani E Rodrigues LT Dellê H Noronha IL Fujihara CK Zatz R Simvastatin attenuates renal inflammation, tubular transdifferentiation and interstitial fibrosis in rats with unilateral ureteral obstruction Nephrol Dial Transplant 2005 20 1582 1591 10.1093/ndt/gfh859 15855201

[B122] Park JK Muller DN Mervaala EM Dechend R Fiebeler A Schmidt F Bieringer M Schäfer O Lindschau C Schneider W Ganten D Luft FC Haller H Cerivastatin prevents angiotensin II-induced renal injury independent of blood pressure- and cholesterol-lowering effects Kidney Int 2000 58 1420 1430 10.1046/j.1523-1755.2000.00304.x 11012877

[B123] Massy ZA Guijarro C Statins: effects beyond cholesterol lowering Nephrol Dial Transplant 2001 16 1738 1741 10.1093/ndt/16.9.1738 11522848

[B124] Sato N Shiraiwa K Kai K Watanabe A Ogawa S Kobayashi Y Yamagishi-Imai H Utsunomiya Y Mitarai T Mizoribine ameliorates the tubulointerstitial fibrosis of obstructive nephropathy Nephron 2001 89 177 185 10.1159/000046065 11549900

[B125] Yoo KH Thornhill BA Forbes MS Coleman CM Marcinko ES Liaw L Chevalier RL Osteopontin regulates renal apoptosis and interstitial fibrosis in neonatal chronic unilateral ureteral obstruction Kidney Int 2006 70 1735 1741 10.1038/sj.ki.5000357 17003824

[B126] Tuck AB Hota C Chambers AF Osteopontin(OPN)-induced increase in human mammary epithelial cell invasiveness is urokinase (uPA)-dependent Breast Cancer Res Treat 2001 70 197 204 10.1023/A:1013095329825 11804183

[B127] Das R Mahabeleshwar GH Kundu GC Osteopontin stimulates cell motility and nuclear factor kappaB-mediated secretion of urokinase type plasminogen activator through phosphatidylinositol 3-kinase/Akt signaling pathways in breast cancer cells J Biol Chem 2003 278 28593 28606 10.1074/jbc.M303445200 12771144

[B128] Musial A Eissa NT Inducible nitric-oxide synthase is regulated by the proteasome degradation pathway J Biol Chem 2001 276 24268 24273 10.1074/jbc.M100725200 11312270

[B129] Broadbelt NV Stahl PJ Chen J Mizrahi M Lal A Bozkurt A Poppas DP Felsen D Early upregulation of iNOS mRNA expression and increase in NO metabolites in pressurized renal epithelial cells Am J Physiol Renal Physiol 2007 293 F1877 F1888 10.1152/ajprenal.00238.2007 17881462

[B130] Hochberg D Johnson CW Chen J Cohen D Stern J Vaughan ED Jr Poppas D Felsen D Interstitial fibrosis of unilateral ureteral obstruction is exacerbated in kidneys of mice lacking the gene for inducible nitric oxide synthase Lab Invest 2000 80 1721 1728 10.1038/labinvest.3780182 11092532

[B131] Manucha W Oliveros L Carrizo L Seltzer A Vallés P Losartan modulation on NOS isoforms and COX-2 expression in early renal fibrogenesis in unilateral obstruction Kidney Int 2004 65 2091 2107 10.1111/j.1523-1755.2004.00643.x 15149322

[B132] Ito K Chen J Khodadadian JJ Seshan SV Eaton C Zhao X Vaughan ED Jr Lipkowitz M Poppas DP Felsen D Liposome-mediated transfer of nitric oxide synthase gene improves renal function in ureteral obstruction in rats Kidney Int 2004 66 1365 1375 10.1111/j.1523-1755.2004.00899.x 15458429

[B133] Chevalier RL Promise for gene therapy in obstructive nephropathy Kidney Int 2004 66 1709 1710 10.1111/j.1523-1755.2004.00984.x 15458470

[B134] Wu JT Kral JG The NF-kappaB/IkappaB signaling system: a molecular target in breast cancer therapy J Surg Res 2005 123 158 169 10.1016/j.jss.2004.06.006 15652965

[B135] Tamada S Asai T Kuwabara N Iwai T Uchida J Teramoto K Kaneda N Yukimura T Komiya T Nakatani T Miura K Molecular mechanisms and therapeutic strategies of chronic renal injury: the role of nuclear factor kappaB activation in the development of renal fibrosis J Pharmacol Sci 2006 100 17 21 10.1254/jphs.FMJ05003X4 16397373

[B136] Goncalves RG Biato MA Colosimo RD Martinusso CA Pecly ID Farias EK Cardoso LR Takiya CM Ornellas JF Leite M Jr Effects of mycophenolate mofetil and lisinopril on collagen deposition in unilateral ureteral obstruction in rats Am J Nephrol 2004 24 527 536 10.1159/000081706 15499219

[B137] Wu MJ Wen MC Chiu YT Chiou YY Shu KH Tang MJ Rapamycin attenuates unilateral ureteral obstruction-induced renal fibrosis Kidney Int 2006 69 2029 2036 10.1038/sj.ki.5000161 16732193

